# Ion Shift Index at the Immediate Post-Cardiac Arrest Period as an Early Prognostic Marker in Out-of-Hospital Cardiac Arrest Survivors

**DOI:** 10.3390/jcm11206187

**Published:** 2022-10-20

**Authors:** Boram Kim, Hyojeong Kwon, Sang-Min Kim, June-Sung Kim, Seung Mok Ryoo, Youn-Jung Kim, Won Young Kim

**Affiliations:** Department of Emergency Medicine, Asan Medical Center, Ulsan University College of Medicine, Seoul 05505, Korea

**Keywords:** ion shift index, out-of-hospital cardiac arrest, prognosis, biomarker

## Abstract

The ion shift index (ISI) is a suggested marker to reflect the magnitude of ischemic damage. This study aimed to investigate the prognostic value of the ISI for predicting poor neurological outcomes at 6 months in comatose out-of-hospital cardiac arrest (OHCA) survivors by comparing it with the OHCA and Cardiac Arrest Hospital Prognosis (CAHP) scores. This observational registry-based cohort study included adult comatose OHCA survivors admitted to a tertiary care hospital in Korea between 2015 and 2021. The ISI was calculated using the serum electrolyte levels obtained within one hour of resuscitation. The primary outcome was poor neurological function (Cerebral Performance Category score of 3–5) at 6 months. Of the 250 OHCA survivors, 164 (65.6%) had poor neurological outcomes. These patients had a higher median ISI than those with good neurological outcomes (4.95 vs. 3.26, *p* < 0.001). ISI (adjusted odds ratio, 2.107; 95% confidence interval, 1.350–3.288, *p* = 0.001) was associated with poor neurological outcomes. The prognostic performance of ISI (area under the curve [AUC], 0.859) was similar to that of the OHCA score (AUC, 0.858; *p* = 0.968) and the CAHP score (AUC, 0.894; *p* = 0.183). ISI would be a prognostic biomarker for comatose OHCA survivors that is available during the immediate post-cardiac arrest period.

## 1. Introduction

Hypoxic-ischemic brain injury is a major complication of out-of-hospital cardiac arrest (OHCA), which leads to about two-thirds of deaths, after intensive care unit admission, of cardiac arrest survivors [1,2]. The active withdrawal of life-sustaining treatment (WLST) for OHCA survivors presumed to survive with a poor neurological outcome accounts for most deaths rather than the direct consequence of loss of brain function [3,4,5]. The current post-resuscitation care guidelines recommend the multimodal neurologic prognostication strategy and postponing the conclusions about the neurological outcome of comatose OHCA survivors until at least 72 h after cardiac arrest; therefore, to minimize premature WLST in patients who might have a chance at neurological recovery [6]. However, the early distinguishment of OHCA survivors presumed to have an irreversible brain injury and anticipated with the minimal benefit of aggressive life-sustaining treatment is essential to avoid futile treatment [6,7].

Recently, many risk scores for supporting emergency clinical decision-making for OHCA survivors, such as the OHCA, MIRACLE2, and Cardiac Arrest Hospital Prognosis (CAHP) scores, were developed; however, they require precise history taking [8,9,10]. Although, cardiac arrest characteristics such as initial cardiac arrest rhythms, collapse time, and the time of initiation of cardiopulmonary resuscitation (CPR) are generally available at the emergency department (ED) admission for OHCA survivors. The reliability of the information is limited due to inaccurate recall or recording, even in the well-established emergency medical service system [8,11]. From that point of view, biomarkers are independent of such bias and are easy to interpret [12,13]. The ion shift index (ISI) has been introduced as a prognostic factor to reflect the magnitude of ischemia damage, defined as the ratio between the concentration of serum intracellular fluid (ICF) ions, i.e., potassium, phosphate, and magnesium, and that of serum extracellular fluid (ECF) ion, i.e., calcium [14,15].

This study aimed to investigate the prognostic value of ISI, during the immediate post-cardiac arrest period, for predicting poor neurological outcomes at 6 months in comatose OHCA survivors and compare the performance to previous prognostic scores, including OHCA and CAHP [8,10].

## 2. Materials and Methods

### 2.1. Study Design and Population

This retrospective, observational, registry-based cohort study was conducted in the emergency department (ED) of a tertiary university-affiliated hospital in Seoul, South Korea. Consecutive adult OHCA patients (aged ≥ 18 years) admitted to the ED have been enrolled in the OHCA registry since 2010, and we extracted the data from this registry [16]. All patients in the registry were followed-up with up to 6 months after ED admission. Their neurological status at 1 and 6 months were assessed using the Cerebral Performance Category (CPC) scores by reviewing the electronic medical records for hospitalized patients or interviewing via a follow-up telephone call with the patient or primary caregiver [16]. The Institutional Review Board of Asan Medical Center approved the study protocol (No. 2022-0743) and waived the need for informed consent due to the retrospective nature of the study.

This study included all consecutive adult patients with successfully resuscitated OHCA, who were admitted to the hospital between January 2015 and December 2021. The excluded patients were those who: (1) were transferred from other hospitals after the return of spontaneous circulation (ROSC); (2) signed advance directives before cardiac arrest; (3) did not undergo the laboratory test within an hour after ROSC; (4) had a poor neurological status prior to cardiac arrest, defined as a CPC of 3 or 4; (5) had chronic renal failure; or (6) refused to participate in the follow-up.

### 2.2. Management and Data Collection

All patients received treatment in accordance with the then-current advanced cardiac life support guidelines [6,17,18,19,20]. During the immediate post-cardiac arrest period, diagnostic studies such as electrocardiography, bedside echocardiography, and computed tomography were performed to identify the cause of OHCA with general intensive care management. The patients received coronary reperfusion and targeted temperature management (TTM) with a target core body temperature of 33 °C or 36 °C using an Arctic Sun Energy Transfer Pad (Medivance Corp., Louisville, CO, USA) if indicated. WSLT was legally prohibited in South Korea until February 2018, and all patients were admitted to hospitals with conservative treatment until death or recovery.

Demographic and clinical data regarding age, sex, previous medical history, presence of a witness on collapse, initial monitored rhythm, arrest cause, time from collapse to CPR, the resuscitation duration, laboratory data during the immediate post-cardiac arrest period, and neurological outcome at 6 months were extracted. For this study, the ISI, OHCA, and CAHP scores were calculated [8,10,14,15]. The ISI was calculated using the following equation:

$$\mathrm{ISI}\;\left(\mathrm{arbitrary}\;\mathrm{unit}\right)=\frac{\mathrm{Potassium}\;\left(\frac{\mathrm{mmol}}{L}\right)+\mathrm{Phosphate}\left(\frac{\mathrm{mmol}}{L}\right)+\mathrm{Magnesium}\left(\frac{\mathrm{mmol}}{L}\right)}{\mathrm{Calcium}\left(\frac{\mathrm{mmol}}{L}\right)}$$

The primary endpoint of this study was a poor neurological outcome at 6 months, defined as a CPC score of 3 (severe cerebral disability), 4 (coma or vegetative status), or 5 (brain death).

### 2.3. Statistical Analysis

Continuous variables were presented as a mean with standard deviations when normally distributed and a median with interquartile ranges when non-normally distributed, based on the Kolmogorov–Smirnov test. Categorical data were presented as absolute numbers with percentages. Comparisons of the characteristics between the good and poor neurological outcome groups were performed using the chi-square test or Fisher’s exact test for categorical variables and the Mann–Whitney U test or Student’s t-test for continuous variables, as appropriate. Univariate logistic analysis was first performed to evaluate the prognostic ability of each variable, and the variables with a *p*-value of <0.10 in the univariate analysis were analyzed by multivariate logistic regression based on a backward elimination method. The results of the logistic regression analysis were summarized using odds ratios (ORs) and the respective 95% confidence intervals (CIs). Variables were tested for goodness of fit using a Hosmer–Lemeshow test. The receiver operating characteristic curves were examined to determine the performance of ISI, OHCA, and CAHP scores in predicting a poor neurological outcome at 6 months [8,10]. The area under the curve (AUC) for each variable was calculated and compared using DeLong’s test [21]. The optimal cutoff value of ISI was determined using the Youden index, which defines the cutoff in terms of the maximal sum of sensitivity and specificity. A two-tailed *p*-value of <0.05 was considered significant. All statistical analyses were performed using IBM SPSS for Windows, version 21.0 (IBM Corp., Armonk, NY, USA) and R (version 3.6.1; R Foundation for Statistical Computing, Vienna, Austria; https://www.R-project.org, accessed on 30 May 2022).

## 3. Results 

**Figure 1:**
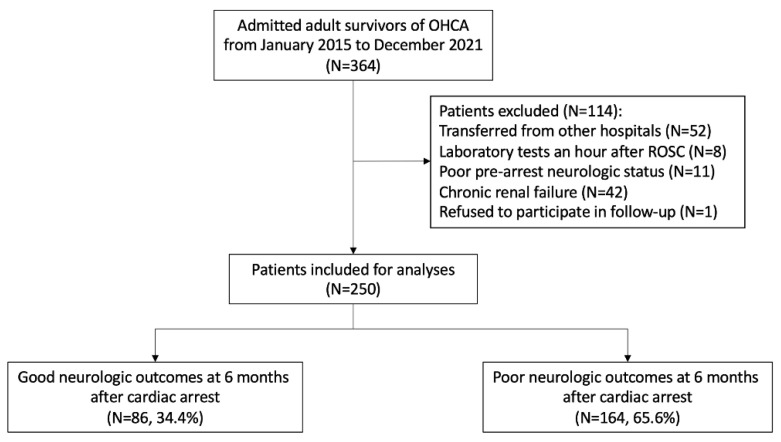
Flow diagram for the enrolment of the patients in the study. Abbreviations: OHCA, out-of-hospital cardiac arrest; ROSC, return of spontaneous circulation.

From January 2015 to December 2021, 364 adult OHCA patients were admitted to the ED of our hospital and received post-cardiac arrest care (Figure 1). Among these cases, 250 patients were finally included in our study after the exclusion of 114 patients who were transferred from other hospitals after the ROSC (*n* = 52), underwent laboratory tests an hour after ROSC (*n* = 8), had a poor pre-arrest neurological status (*n* = 11), had chronic renal failure (*n* = 42), or refused to follow-up (*n* = 1). A total of 164 patients (65.6%) in this cohort had a poor neurological outcome (CPC score ≥ 3) at 6 months after ROSC.

The baseline characteristics and laboratory results are presented in Table 1. Patients with poor neurological outcomes were older (median, 55.6 vs. 64.0 years, *p* = 0.001) and had a lower rate of witnessed cardiac arrest (88.4% vs. 71.3%, *p* = 0.002) and initial shockable rhythm (70.9% vs. 23.8%; *p* < 0.001). Total collapse time and resuscitation duration were longer in the poor neurological outcome group, while the no flow time did not significantly differ between the two groups. All laboratory results during the immediate post-cardiac arrest period significantly differed between the two groups; the concentrations of serum ICF ions, including potassium, phosphate, and magnesium, were higher in the poor neurological outcome group, while that of the serum ECF, i.e., calcium, was lower. The proportion of TTM did not differ significantly between the patients with good and poor neurological outcomes (77.9% vs. 84.1%, *p* = 0.223). In patients with a poor neurological outcome, the ISI was higher than that of those with a good neurological outcome (median, 3.26 vs. 4.95, *p* < 0.001), as were the OHCA (mean, 21.35 vs. 41.24, *p* < 0.001) and CAHP (median, 118.35 vs. 203.62, *p* < 0.001) scores.

**Figure 2:**
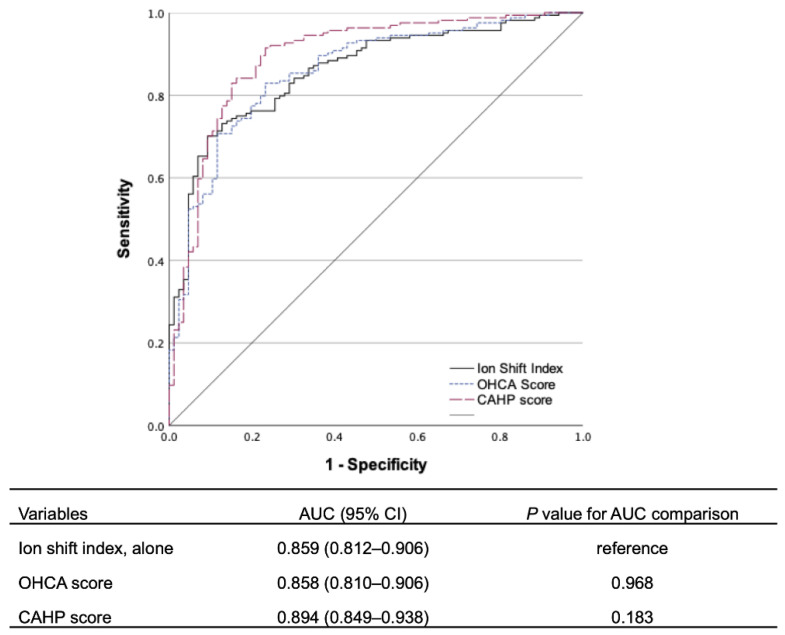
Comparison of the prognostic performances of ion shift index, out-of-hospital cardiac arrest (OHCA), and Cardiac Arrest Hospital Prognosis (CAHP) scores for predicting a poor neurological outcome at 6 months. Abbreviations: AUC, area under the curve; CI, confidence interval.

In the univariate logistic regression analysis, age, previous diabetes mellitus, unwitnessed collapse, initial non-shockable rhythm, arrest cause, resuscitation duration, pH, lactate levels, and ISI were associated with a poor neurological outcome at 6 months (Table 2). In multivariate logistic regression analysis, ISI (adjusted OR, 2.107; 95% CI, 1.350–3.288; *p* = 0.001) is associated with poor neurological outcomes at six months. The AUC for ISI was 0.859 (95% CI, 0.812–0.906), similar to that of the OHCA (0.858; 95% CI, 0.810–0.906; *p* = 0.968) and CAHP (0.894; 95% CI, 0.849–0.938; *p* = 0.183) scores (Figure 2).

The optimal cutoff value for ISI, based on the Youden index, was 4.25, with a 70.1% sensitivity and a 90.7% specificity (Table 3). An ISI-value of >6.40 predicted a poor neurological outcome at 6 months with a 0% false positive rate and a 24.4% sensitivity.

## 4. Discussion

This registry-based cohort study demonstrated that ISI during the immediate post-cardiac arrest period was independently associated with a poor neurological outcome at 6 months, which implied that ISI could be used as a prognostic biomarker available within an hour after ROSC. Without other clinical factors, ISI, using the serum concentration level of potassium, phosphate, magnesium, and calcium, showed similar prognostic performance for a 6-month neurological outcome in comatose OHCA survivors compared to the OHCA and CAHP scores. Considering that ISI is an objective prognostic biomarker that does not rely on memory and records, it could be useful for OHCA survivors during the immediate post-cardiac arrest period, especially when combined with other predictive clinical features.

Electrolyte derangement is common in cardiac arrest survivors. Ischemic injury due to cardiac arrest alters the energy-dependent membrane-bound ionic pumps and results in cell membrane dysfunction and homeostasis loss. The efflux of potassium and magnesium ions from the cytoplasm and rapid phosphate production from the adenosine triphosphate breakdown occurs in the early phase of hypoxia without the inhibition of the sodium-potassium adenosine triphosphatase pump and the absence of cell injury. This is followed by the influx of sodium, chloride, and calcium ions and a further loss of cytoplasmic potassium and magnesium in accord with the ionic pump dysfunction and cellular injury [22,23]. The cell swelling and electrolyte derangements are evident even in the reversible phase of cell injury. The high permeability of the cellular membrane in the irreversible phase of cell injury results in the influx of extracellular calcium and exacerbates damage to the mitochondria and cytoskeleton, leading to cell death [23].

Previous studies revealed that levels of even a single electrolyte, including potassium and phosphate, had a significant association with the outcome in cardiac arrest survivors [24,25]. Lee et al. hypothesized that the ISI would reflect the degree of cellular damage and demonstrated that a higher ISI was associated with poor neurological outcomes at discharge in 580 in-hospital and out-of-hospital cardiac arrest survivors (adjusted OR, 2.916; 95% CI, 1.798–4.730) with an AUC of 0.878 [14]. Consistent with this previous study, our study also demonstrated that a higher ISI was associated with a poor neurological outcome at 6 months in comatose OHCA survivors (adjusted OR, 2.107; 95% CI, 1.350–3.288) with an AUC of 0.859.

Many risk stratification tools for OHCA survivors in the immediate post-cardiac arrest period have been developed for better treatment strategies and allocations of medical resources [8,10,26]. In this study, the prognostic performance of ISI (AUC, 0.859) was similar to other risk stratification scores including the OHCA (0.858; 95% CI, 0.810–0.906; *p* = 0.968) and CAHP (0.894; 95% CI, 0.849–0.938; *p* = 0.183) scores in predicting the poor neurological outcome of OHCA survivors. Compared to these clinical scores, ISI is easier to calculate and can be used in emergencies independent of clinical information. Several established clinical variables used in the OHCA and CAHP scores, including age, a witnessed arrest, initial rhythm, resuscitation duration, and pH, were also significantly associated with outcome in this cohort. Combining ISI with these clinical factors would result in an enhanced prognostic performance, which suggests that ISI is a potential biomarker to be incorporated into future studies to establish optimal treatment strategies for OHCA patients in immediate post-resuscitation duration.

This study has several limitations. First, this study was a retrospective, observational, registry-based cohort study performed in a single center, which limited the generalizability of the results and made the presence of unmeasurable confounding bias inevitable. Second, this study included OHCA patients between 2015 and 2021, and the treatment guidelines have been since updated [6,17,18,19,20]. This could affect the clinical decision-making of the physician and, consequently, the outcome for OHCA survivors. In addition, not all patients underwent TTM, which could affect the outcome. Third, this study excluded 42 patients (11.5% from the registry) diagnosed with chronic renal failure before OHCA, which might cause a selection bias. However, patients with chronic renal failure might have electrolyte derangement, such as hyperkalaemia, before cardiac arrest, and therefore the ISI of such patients would not purely reflect the degree of the cell injury after cardiac arrest [27,28]. In addition, other medical conditions such as endocrine disorders or medications such as furosemide, indapamide, and spironolactone could result in the pre-arrest electrolyte imbalance and would interfere with the ISI performance. Finally, the withdrawal of life-sustaining treatments (WLST) or death after awakening would confound the 6-month neurological outcome. In our cohort, there was no premature WLST during targeted temperature management, and the WLST was illegal before February 2018 in South Korea, indicating the low risk of self-fulfilling prophecy bias.

## 5. Conclusions

This study validated that the ISI reflects the degree of cell damage in OHCA survivors, and its use alone (without other added measures) showed a similar prognostic performance to other risk stratification scores. ISI is a potential objective prognostic biomarker for predicting neurological outcomes, which does not rely on memory and records and can be easily applied during the immediate post-cardiac arrest period. Further studies incorporating ISI into other variables for predicting neurological outcomes in OHCA survivors are warranted.

## Figures and Tables

**Table 1:** Demographic and clinical characteristics of the patients with out-of-hospital cardiac arrest.

Characteristics	Total (*n* = 250)	Patients with Good Neurological Outcomes at 6 Months (*n* = 86)	Patients with Poor Neurological Outcomes at 6 Months (*n* = 164)	*p* Value
Age, years	62.0 (50.0–70.3)	56.5 (47.8–66.0)	64.0 (50.3–73.8)	0.001
Number of males	180 (72.0%)	67 (77.9%)	113 (68.9%)	0.132
Comorbid disease				
Hypertension	89 (35.6%)	30 (34.9%)	59 (36.0%)	0.864
Diabetes Mellitus	47 (18.8%)	11 (12.8%)	36 (22.0%)	0.078
Witnessed cardiac arrest	193 (77.2%)	76 (88.4%)	117 (71.3%)	0.002
Bystander CPR	179 (71.6%)	66 (76.7%)	113 (68.9%)	0.191
Initial shockable rhythm	100 (40.0%)	61 (70.9%)	39 (23.8%)	<0.001
Arrest cause				<0.001
Presumed cardiac	148 (59.2%)	74 (86.0%)	74 (45.1%)	
Other medical	63 (25.2%)	6 (7.0%)	57 (34.8%)	
External *	39 (15.6%)	6 (7.0%)	33 (20.1%)	
Total collapse time, min	25.0 (12.0–41.0)	14.5 (8.0–27.0)	33.5 (16.3–46.0)	<0.001
No flow time, min	0.0 (0.0–4.0)	0.0 (0.0–3.0)	0.0 (0.0–5.0)	0.278
Resuscitation duration, min	23.0 (11.0–37.0)	13.0 (7.0–23.8)	30.0 (16.0–44.0)	<0.001
Laboratory results				
Potassium, mmol/L	4.2 (3.6–5.2)	3.9 (3.4–4.2)	4.6 (3.8–5.8)	<0.001
Phosphorous, mmol/L	2.39 (1.84–3.01)	1.89 (1.53–2.30)	2.68 (2.11–3.35)	<0.001
Magnesium, mmol/L	1.05 (0.94–1.17)	0.96 (0.89–1.04)	1.10 (1.00–1.21)	<0.001
Calcium, mmol/L	2.15 (2.00–2.27)	2.20 (2.08–2.31)	2.13 (1.98–2.25)	0.011
pH	7.02 (6.86–7.19)	7.22 (7.09–7.30)	6.92 (6.80–7.07)	<0.001
Lactate, mmol/L	11.2 (7.4–14.3)	7.8 (5.2–11.0)	12.6 (9.4–15.0)	<0.001
Targeted temperature management	205 (82.0%)	67 (77.9%)	138 (84.1%)	0.223
Ion shift index	4.21 (3.37–5.51)	3.26 (2.69–3.74)	4.95 (3.93–6.32)	<0.001
OHCA score	34.39 (16.23)	21.35 (14.00)	41.24 (12.78)	<0.001
CAHP score	187.47 (131.02–216.78)	118.35 (96.37–148.89)	203.62 (180.11–229.88)	<0.001

* The external cause of cardiac arrest included hanging (*n* = 15), suffocation (*n* = 9), drowning (*n* = 6), trauma (*n* = 5), and poisoning (*n* = 4). Values are expressed as median (interquartile ranges), mean (standard deviation), or *n* (%) as appropriate. Abbreviations: CAHP, Cardiac Arrest Hospital Prognosis; CPR, cardiopulmonary resuscitation; OHCA, out-of-hospital cardiac arrest.

**Table 2:** Logistic regression analysis for a 6-month poor neurological outcome in the patients with out-of-hospital cardiac arrest.

Characteristics	OR	95% CI	*p* Value	Adjusted OR	95% CI	*p* Value
Age, years	1.026	1.009–1.044	0.003	1.044	1.014–1.076	0.004
Male	0.628	0.392–1.153	0.134			
Comorbid disease						
Hypertension	1.049	0.607–1.811	0.864			
Diabetes Mellitus	1.918	0.921–3.991	0.082			
Unwitnessed	3.053	1.455–6.406	0.003	2.557	0.821–7.966	0.105
No bystander CPR	1.489	0.818–2.713	0.193			
Initial non-shockable rhythm	7.821	4.343–14.081	<0.001	2.895	0.993–8.444	0.052
Arrest cause						
Presumed cardiac	Reference		<0.001	Reference		0.110
Other medical	9.500	3.859–23.385	<0.001	3.941	1.053–14.747	0.042
External *	5.500	2.175–13.907	<0.001	2.709	0.574–12.774	0.208
No flow time, min	1.048	0.984–1.115	0.147			
Resuscitation duration, min	1.054	1.033–1.075	<0.001	1.029	1.003–1.057	0.030
pH	0.001	0.000–0.006	<0.001	0.021	0.002–0.237	0.002
Lactate, mmol/L	1.298	1.198–1.406	<0.001			
Ion shift index	3.753	2.579–5.463	<0.001	2.107	1.350–3.288	0.001
Targeted temperature management	1.505	0.778–2.911	0.224			

* The external cause of cardiac arrest included hanging (*n* = 15), suffocation (*n* = 9), drowning (*n* = 6), trauma (*n* = 5), and poisoning (*n* = 4). Abbreviations: CI, confidence interval; CPR, cardiopulmonary resuscitation; OR, odds ratio.

**Table 3:** Ion Shift Index cutoff values in predicting a poor neurological outcome at 6 months.

Type	Cut-Off Value	Sensitivity	Specificity	Number of Patients			
				True Positive	True Negative	False Positive	False Negative
0% FNR	2.07	100.0%	5.8%	164	5	81	0
Youden index	4.25	70.1%	90.7%	115	78	8	49
<5% FPR	4.71	56.1%	95.3%	92	82	4	72
0% FPR	6.40	24.4%	100.0%	40	86	0	124

Abbreviations: FNR, false negative rate; FPR, false positive rate.

## Data Availability

The data used to support the findings of this study are available from the corresponding author upon request.
